# A125 A NOVEL TECHNIQUE COMBINING EUS AND SPYGLASS DS SYSTEM GUIDANCE FOR BOTH THE DIAGNOSIS AND MANAGEMENT OF AFFERENT LIMB SYNDROME

**DOI:** 10.1093/jcag/gwab049.124

**Published:** 2022-02-21

**Authors:** F Almousawi, B Kim, J Mosko

**Affiliations:** Gastroenterology, University of Toronto, Toronto, ON, Canada

## Abstract

**Background:**

Afferent limb syndrome is obstruction of a biliary-enteric limb following pancreaticoduodenectomy. In the past, treatment was limited to surgery or insertion of a percutaneous transhepatic biliary drainage tube. A more recent approach to treatment is Endoscopic Ultrasound (EUS)-guided gastrojejunostomy using a lumen apposing metal stent (LAMS). It was first described in two single case reports in 2015 and has been growing in its application. We present a case series of 2 patients where afferent limb syndrome was successfully managed with EUS guided gastrojejunostomy using LAMS.

**Aims:**

1-To demonstrate a novel technique for diagnosis and management of afferent limb syndrome.

**Methods:**

Case # 1- 79 year old female post Whipple’s surgery for ampullary carcinoma, presented two years after surgery with significant nausea and vomiting. CT scan showed obstruction of the afferent loop suspicious for local tumor recurrence. Patient underwent EUS-guided gastrojejunostomy with successful insertion of 20 x 10 mm (AXIOS EC, Boston Scientific) stent.

Case # 2-. A 51 year old male with pancreatic CA status post Whipple surgery presented with worsening abdominal pain and nausea. CT scan confirmed afferent limb syndrome. He underwent EUS- guided gastrojejunostomy with successful placement of 10 x 10 mm (AXIOS EC, Boston Scientific).

**Results:**

Case #1: Patient improved dramatically within days and resumed oral diet. Fourteen days later, an upper endoscopy was performed in order to assess the stricture via an anterograde approach. The adult Olympus HQ gastroscope was advanced through the AXIOS stent into the afferent limb to the level of the stenosis where a tight stricture was seen. Multiple biopsies taken and were negative for malignancy. Four weeks later, she continued to tolerate oral diet well with no abdominal pain or discomfort.

Case # 2: Patient continued to do well clinically 12 weeks post operation. Follow-up upper endoscopy was performed to determine the etiology of the obstruction but due to a sharp angulation, the gastroscope could not be advanced through the AXIOS stent. As such, we advanced an adult Olympus 1T gastroscope down to the site of the AXIOS stent and then utilized the Spyglass DS system to advanced deep into the afferent limb to the level of the stricture and took multiple biopsies for diagnostic purposes.

**Conclusions:**

Interventional endoscopic management of afferent limb syndrome has been evolving in the past few years with EUS guided LAMS gastrojejunostomy becoming a well stablished modality. We have demonstrated this technique with subsequent endoscopic anterograde assessment. In one of these cases, this anterograde assessment was performed using the cholangioscope in conjunction with the gastroscope to overcome unfavorable angulated anatomy which to our knowledge has not been described in the literature previously.

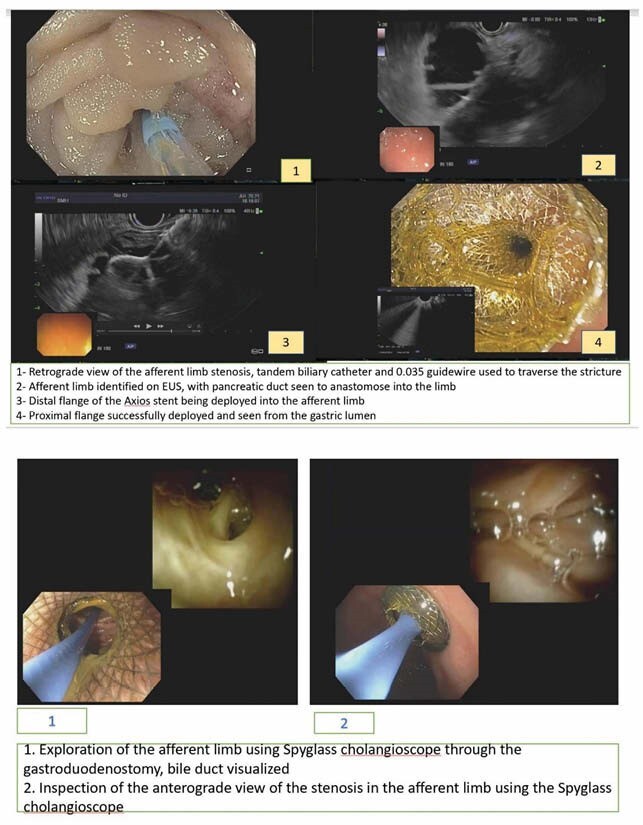

**Funding Agencies:**

None

